# Fish grabbing: Weak governance and productive waters are targets for distant water fishing

**DOI:** 10.1371/journal.pone.0278481

**Published:** 2022-12-06

**Authors:** Moritz Stäbler, Jonas Letschert, Marie Fujitani, Stefan Partelow

**Affiliations:** 1 Leibniz Centre for Tropical Marine Research (ZMT), Bremen, Germany; 2 Thuenen Institute of Sea Fisheries, Braunschweig, Germany; 3 University of Bremen, Bremen, Germany; University of New Haven, UNITED STATES

## Abstract

Distant water fishing occurs worldwide as foreign fleets fish in the exclusive economic zones (EEZs) of other states. We test the hypothesis that host state governance performance is an explanatory factor in observed distant water fishing effort using Global Fishing Watch’s fishing effort data obtained from vessels’ automatic identification system (AIS). We examine the explanatory power of the World Governance Indicators (WGI), Gross Domestic Product (GDP) per capita, and biophysical fisheries productivity indicators (temperature, oxygen, salinity, nutrients, and primary productivity) on fishing effort from foreign fleets across the four most common gear types (fixed gear, longliners, trawlers, and tuna purse seiners). Our models include both host EEZ fishery productivity indicators and governance indicators with R^2^ values of 0.97 for longlining, 0.95 trawling, 0.95 for fixed gear and 0.82 for tuna purse seiners. Although a lack of good governance may enable illegal, unreported and unregulated fishing, the United Nations Convention of the Law of the Sea (UNCLOS) has enabled the legal establishment of foreign fishing contracts. However, it is unlikely that fishing contracts are decoupled from economic and political negotiations on other issues. We argue that it is worthwhile to consider the term “fish grabbing”, meaning wealthier and politically more powerful states consciously seek to profit from fishing in the waters of often weaker states through developing legal fishing contracts.

## 1.0 Introduction

Many countries fish in the waters of other states [1–6], conducting distant water fishing (DWF). We define DWF as when foreign fleets fish in another country’s EEZ, by either negotiating a contract, which may be between governments and/or private companies (e.g., joint ventures, foreign access agreements, licensing) [7], or fishing illegally [8]. One example would be a Chinese or Spanish vessel fishing within the EEZ of Ghana (i.e., with a contract or illegally). In the general capture fisheries literature, the impact of social, economic, and political factors in shaping the distribution of fishing effort has been extensively explored [8–13]. For example, Österblom et al., [11] make the important connection between weak governance of flag (sending) states and IUU fishing. Gutiérrez et al., [14] focus on China, with the largest global DWF fleet, analyzing the extent and impact of their fleets around the world, while Collins et al., [15] show on a smaller scale the social-ecological drivers of Sri Lankan offshore fleets. McCauley et al., (2018) further show the dominance of wealthy nations. However, specific to DWF, no studies have yet examined the governance performance of host states in relation to the amount of received DWF effort, a hypothesis that challenges a long standing paradigm in fisheries research that the distribution of fishing effort is driven primarily by fisheries productivity [16, 17]. In this study, we use random forest models to test the importance of host state governance performance in explaining effort distribution using Global Fishing Watch data based on the Automatic Identification System (AIS) data. Our findings allow us to further explore the likelihood that certain vessel flag states prioritize DWF contracts with countries with weaker governance and economic indicators. We introduce the term ‘fish grabbing’, meaning wealthier and politically more powerful states consciously seek to profit from fishing in the waters of often weaker states through developing legal fishing contracts, or through fishing illegally.

Offshore resource management and use rights were given to states under the United Nations Convention of the Law of the Sea (UNCLOS) within their Exclusive Economic Zone (EEZ) up to 200 nautical miles offshore [18]. Article 2 of the Convention [18] states that, “the sovereignty of a coastal State extends, beyond its land territory and internal waters…”, continuing more specifically in Article 61, that “the coastal State shall determine the allowable catch of the living resources in its exclusive economic zone.” Within this context, a wide range of international partnerships, regulatory mechanisms and trade agreements have been established to help specifically mitigate illegal, unregulated and unreported fishing (IUU) by distant water fleets [19]. Nonetheless, legal fishing contracts are likely to be interconnected with economic and political negotiations on other issues [20–25], where power, in the form of economic and political leverage, held by nations doing the distant water fishing can lead to disproportionate gains for them and uncompensated environmental, livelihood and food security losses for host states (i.e., those being fished).

While there is an established ‘grabbing’ literature examining the economic and political power disparities that can exist within foreign land and freshwater acquisitions between wealthier resource-seeking states and less wealthy resource-abundant states [26–28], these potential disparities have yet to be critically explored with substantive empirical data in relation to purposeful DWF activities [7]. They have, however, been extensively examined in the general fishing and broader ocean resource use literature [29, 30], such as on community conservation [31], seabed mining [32] and small-scale fishing [33]. One reason that DWF has not been examined through a grabbing lens, despite the clear potential for illegal or contract fishing to be intertwined with neocolonial and neoimperial interests, is that high-resolution data on fishing effort has been historically scarce before the systematic collection of Automatic Identification System (AIS) data to extract fishing effort by Global Fishing Watch [17, 34]. Additionally, the terms of foreign fishing contracts are often unknown, but the few existing studies on fishing contract agreements provide support for the need to further examine if, how and why ‘fish grabbing’ exists [21, 22, 24]. For example, foreign fishing contracts could provide equitable benefits to both host countries and distant water fishers in cases where a “proper redistribution of the gains from trade and proper management of the resources are in place” (p. 182) [24]. However, evidence suggests that most fishing contracts create unfair terms that benefit developed country DWF fleets and disadvantage developing countries [7, 21, 35, 36]. In some cases, contracts do not exist at all or are not followed [14, 37]. If the host state has a developing economy, it likely also has less political stability and less established science systems for fisheries assessments [38]. Not being able to assess resource population status, value or ecosystem health accurately makes sustainable resource management challenging, further making it unlikely that agreed upon DWF contracts ensure sustainability and fair economic gains. Nonetheless, some host states have decided to focus on mitigating illegal DWF fishing in their waters, as shown by Cabral et al., [39] in Indonesia, which reduced foreign effort without incurring losses of domestic fishing effort reductions.

In this article, we examine if host state governance performance influences DWF effort and foreign access to fishing within their EEZ, while also considering environmental and fishery productivity indicators within their EEZ. We specifically test the hypothesis that host state governance performance indicators are relevant drivers for DWF patterns and discuss whether host states with lower governance performance receive more fishing effort from foreign fleets than would be expected given their level of biophysical productivity. EEZs of each country are our units of analysis for fishing behavior, for which we aggregate DWF effort data (Global Fishing Watch), governance performance (World Governance Index) and economic indicators (GDP per capita), as well as biophysical parameters important for fisheries productivity. Given the heterogeneity of factors related to gear types, including target species, fishing behavior, geographic extent, and seasonality [10, 40], we undertake separate analyses for the four most common gear types in DWF: longliners, trawlers, fixed gear and purse seiners.

## 2.0 Methods

### 2.1 Fishing effort data

We used Global Fishing Watch (GFW) data representing daily gridded fishing effort on the 100^th^ degree latitude and longitude in 2017, being the highest quality data and most recent available at the time of data analysis (www.globalfishingwatch.org; accessed 01.03.2019) [17]. We decided to only use fishing effort from one year, since data quality and coverage increases with time complicating comparisons across years. The data set contains the following information per grid cell and day: fishing effort in hours, flag state, gear type, latitude, and longitude. For each grid cell we determined the coordinates of the 4 corner points and identified whether these are located in an EEZ, in areas beyond national jurisdiction, on land, or in multiple of these categories. To distinguish between EEZ or land area we used shape files from marine regions (www.marineregions.org; accessed 07.05.2018). We were able to identify the majority of the corner points of all grid cells in the data set. Remaining unidentified points were located between shapefiles and were manually assigned to one of the mentioned categories. The *R* language for statistical computing was used for all data related tasks [41]. We aggregated fishing effort by flag state (sending country), gear and EEZ (host country). Distant water fishing (DWF) was then defined as fishing activities undertaken by vessels registered with a country (flag) different from the EEZ they are fishing in. All domestic fishing data (i.e., the vessel flag matches the EEZ state) were removed. We also removed all intra-EU fishing, because EU fisheries is managed across EEZ under the common fisheries policy (CFP) enabling access without fishing agreements. For the sake of this study, we treated Norway like an EU member state due to agreements with the EU regulating the mutual exploitation of shared fish stocks. Furthermore, we removed EEZs with disputed claims and those under commonly managed regimes or state territories. A list of all EEZs this applied to can be found in the supplementary material (S1 Table in S1 File). Due to the very unequal sizes of EEZs worldwide (Fig A1 in S1 File), we standardized DWF by dividing it by the size of the EEZ in km^2^.

### 2.2 Economic, governance, and biophysical data

Prior to modelling, and per data source, the data was centered and scaled (z-score standardized), and missing values were imputed using k-Nearest Neighbor algorithms (knn). Gross Domestic Product (GDP) per capita data in 2017 USD [42] was used as an economic performance indicator. This was matched to each EEZ, along with data on the six governance performance indicators from the World Governance Indicators (WGI), namely: control of corruption, government effectiveness, political stability (no violence), regulatory quality, rule of law, and voice and accountability [43] (https://info.worldbank.org/governance/wgi/). These six WGI indicators of broad dimensions of governance cover over 200 countries and have been collected since 1996. WGI indicators were only available for sovereign states, but not for state territories, such as American Samoa (state territory of USA) or Christmas Island (state territory of Australia). State territories often have very different political and economics standards than their sovereign states, which is why using sovereign state WGI indicators for state territories would have obscured our analysis. Therefore, we removed state territories from our analysis. A list of all removed state territory EEZs (S1 Table in S1 File), a summary of all data categories used in this study, along with their sources, and specific variables (S2 Table in S1 File), as well as global maps of governance and biophysical data distributions per EEZ (Figs A2 and A3 in S1 File) are provided in the supplementary material. Detailed definitions of each of the six WGI dimensions are provided in Table 1, along with interpretative justifications for the relevance of each individual indicator to fishing related policies and governance.

**Table 1:** World governance indicator definitions, and potential relevance for DWF.

Indicator	Definition (Kaufmann et al., 2011)	Hypothesized relevance for DWF
Control of Corruption	the extent to which public power is exercised for private gain, including both petty and grand forms of corruption, as well as "capture" of the state by elites and private interests.	In the absence of strong institutions, elites could perform ‘fish grabbing’ from their own citizens, and sell rights under unfavorable terms which benefit elites but disadvantage citizens’ dependent on those fishery resources for livelihoods and food security.
Government Effectiveness	the quality of public services, civil service and the degree of its independence from political pressures, the quality of policy formulation and implementation, and the credibility of the government’s commitment to such policies.	Effective government can avoid unfair contracts through implementation of sound fisheries policy programs and avoiding economic and political power dependencies on perhaps wealthier foreign fishing entities.
Political Stability (No Violence)	the likelihood that the government will be destabilized or overthrown by unconstitutional or violent means, including politically‐motivated violence and terrorism.	The ability of continuity and consistency in fisheries policy that uphold UNCLOS commitments, while avoiding dependency on foreign economic and political capital through steady domestic development.
Regulatory Quality	the ability of the government to formulate and implement sound policies and regulations that permit and promote private sector development.	Ability to develop sound fisheries policy, monitoring and sanctioning that create reliable data and management frameworks. Known quality may deter illegal fishing.
Rule of Law	the extent to which agents have confidence in and abide by the rules of society, and in particular the quality of contract enforcement, property rights, the police, and the courts, as well as the likelihood of crime and violence.	Belief in the effectiveness and quality of host state governance, can influence the willingness of DWF states to conduct illegal activities or develop knowingly unfair agreements, through fear of sanctioning.
Voice and Accountability	the extent to which a country’s citizens are able to participate in selecting their government, as well as freedom of expression, freedom of association, and a free media.	Citizens influence and expression rights over concerns of governance corruption, effectiveness and quality can avoid elite capture and provide a feedback from observed or felt negative effects of overfishing on, for example, livelihoods dependent on local marine resources.

As proxies of fisheries productivity in each EEZ, we used modelled primary productivity (PP) data from the Seas Around Us project (http://www.seaaroundus.org/sea-around-us-area-parameters-and-definitions/#_Toc421807913). Note that PP was assigned per sovereign EEZ, and that for states with multiple EEZs (e.g., Canadian Pacific and Atlantic coast), the respective largest EEZ served as reference to represent the respective area with the most fishing effort. We sourced biophysical data from the NOAA World Ocean Atlas, consisting of decadal averages at sea surface level (SSL) and at 50 m depth. For each EEZ, we computed mean annual water temperature, salinity, oxygen concentration, silicate, and nitrate. To ensure a sufficiently large sample size, we only included gear types with DWF occurring in at least 40 EEZs (Fig A4 in S1 File). Due to missing PP data, we removed the Monegasque EEZ from our analysis.

### 2.3 Model building and comparison

Gear by gear, we modelled DWF effort per EEZ [h/km^2^] as a function of the EEZs’ average primary productivity, salinity, oxygen concentration, silicate, nitrate, and phosphate; each at the surface level and in 50m depth, as well as the EEZ sovereigns’ GDP, control of corruption, government effectiveness, political stability, regulatory quality, rule of law, and voice and accountability.

Three alternative model types were tested for their best performance in predicting DWF fishing effort: an elastic-net regularized Generalized Linear Model (R package: glmnet) [44], a Random Forest model (RF; R package: ranger) [45], and a Generalized Boosted Regression Model (R package: gbm) [46]. Model performance (as lowest RMSE) was evaluated using 10-fold cross validation with custom folds to ensure the cross validation was always undertaken on the same subsamples. Per gear, the final model of the best performing model family was then explored for its predictions. RF models outperformed the predictions of Generalized Boosted Regression Models and Elastic-net GLMs in the case of all four gear groups examined (Fig A6 in S1 File).

We used variable importance (VI) scores, a relative measure indicating how often a variable was used for a split in the final RF models, to identify the relevance of variables at explaining fishing effort. We considered variables to be meaningful (informative) if they exceeded the VI score of a random number, which we added as an additional predictor to each modelling case [47]. For those variables deemed informative, we used Accumulated Local Effects (ALE) plots [48] (R package: ALEPlot) [49] to assess how they affect the final models’ prediction of DWF effort.

## 3.0 Results

**Fig 1:**
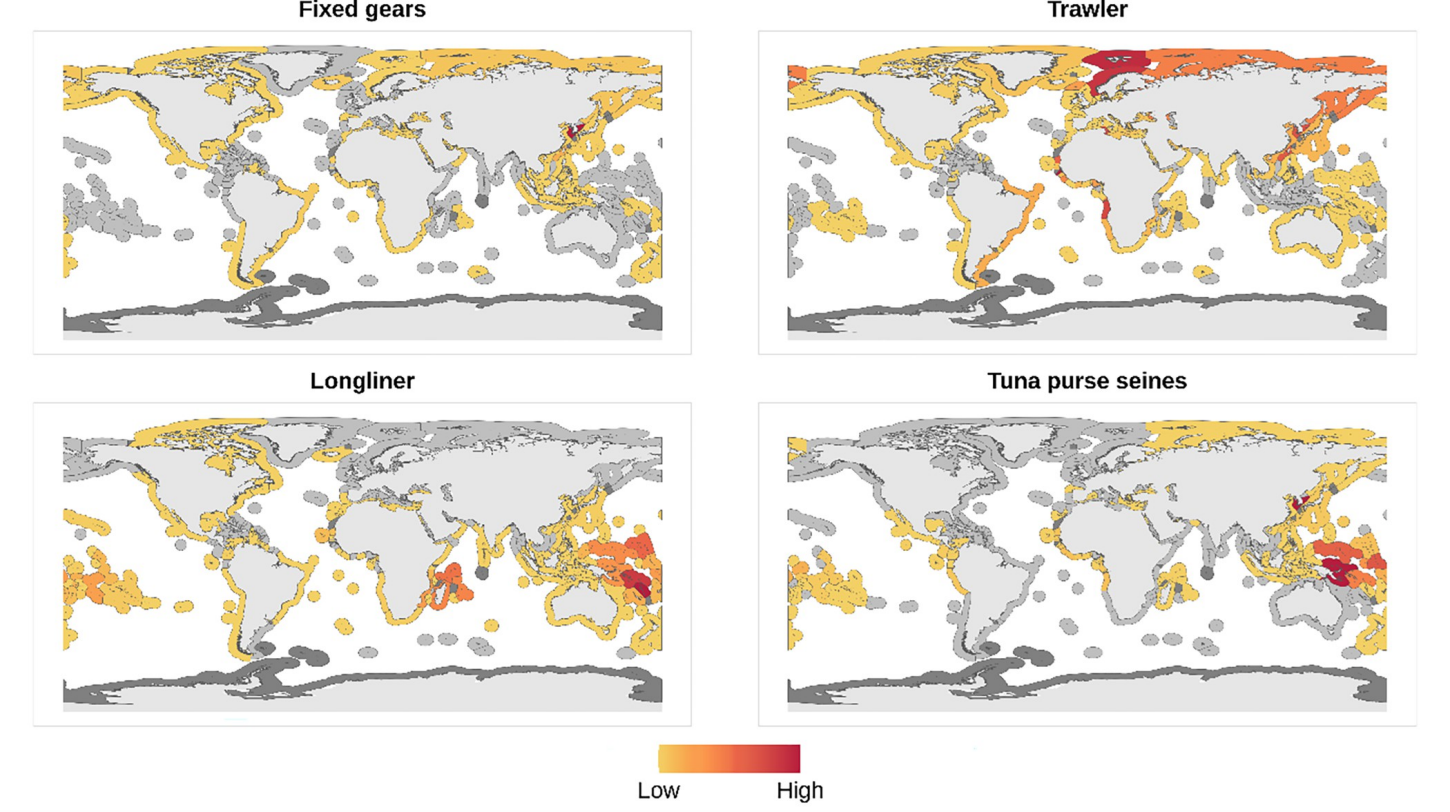
Standardized distant water fishing hours per exclusive economic zone (EEZ). The panels display the four fishing gears used in this study. EEZs without any distant water fishing effort in the year 2017 are represented in light grey (e.g., Somalia) and those without or shared sovereignty and disputed claims are represented in dark grey (e.g., Antarctica). Note that due to missing governance indicators for state territories, we did consider these in our statistical analysis (see S2 Table in S1 File).

Our calculation of standardized DWF hours per km^2^ shows high spatial variation based on gear type across EEZs, with observable regional patterns (Fig 1). Fixed gear DWF effort is highest in the South Korean EEZ followed by Taiwan. There is none or very little fixed gear DWF effort in the Caribbean and Pacific Island states. Trawler DWF effort is highest, in order, off Guinea Bissau, Svalbard, Norway, Angola, Tunisia, South Korea, Taiwan, Mauritia, Russia, Sierra Leone, Congo and Nigeria. Longlining DWF effort is highest off Madagascar, Micronesia, and the Marshall Islands, and generally higher in the east and central Pacific. Tuna purse seiner DWF shows a similar pattern of high effort in the east and central Pacific, specifically in the South Korean and Papua New Guinean EEZs. Across all four gear types, hotspots of DWF hours fished per km^2^ are all in tropical regions.

**Fig 2:**
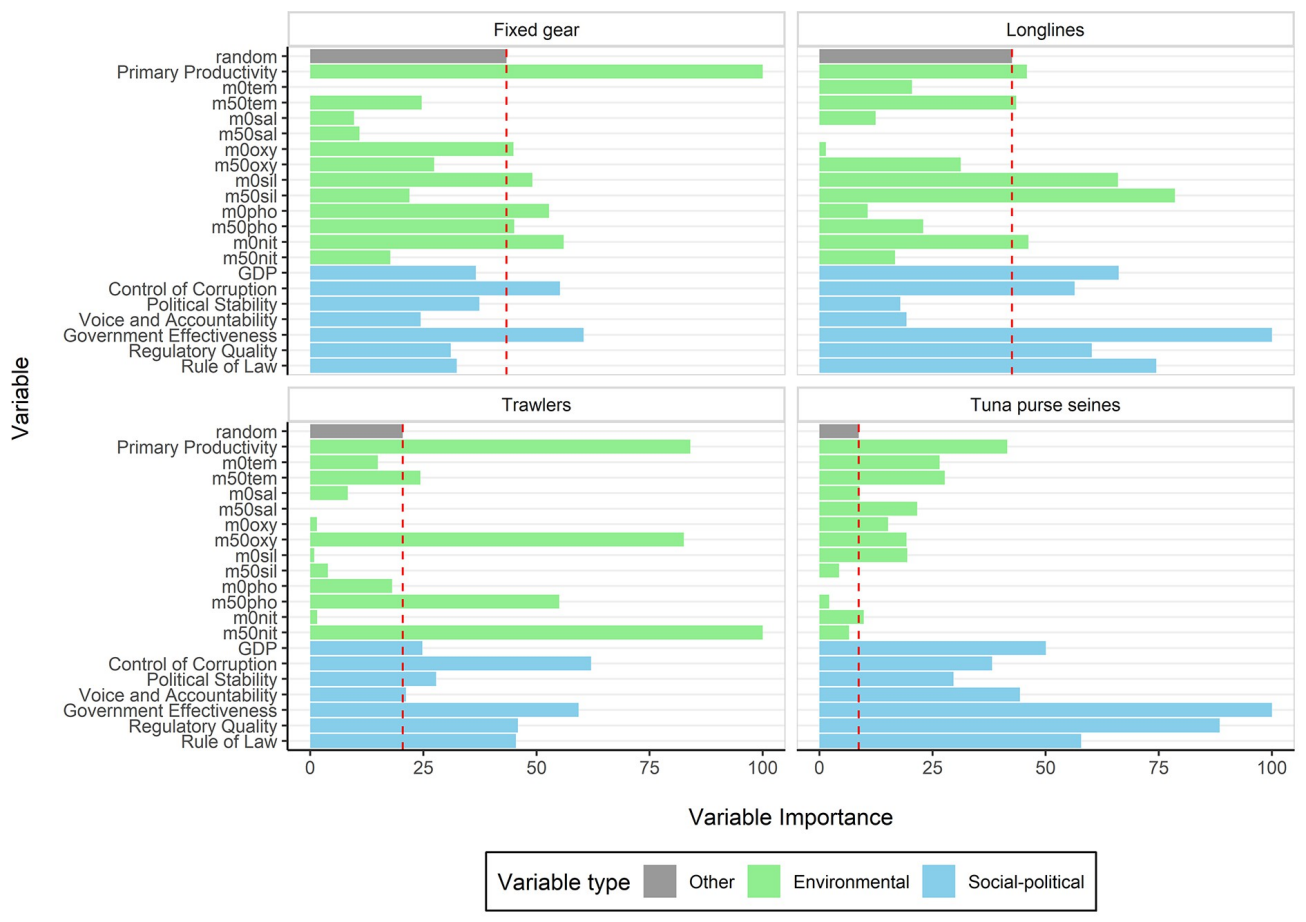
Variable importance (VI) scores of environmental and socio-political variables for the four fleets investigated, each relative to the respective highest VI. We defined variables with a higher VI than that of a random variable (red dotted line) as informative.

Our random forest models, predicting DWF effort with biophysical indicators and governance parameters, show high explanatory power across all gear types of DWF with R^2^ values of 0.95 (trawlers), 0.97 (longliners), 0.95 (fixed gear) and 0.82 (tuna purse seiners). Informative variables, which are more important in predicting DWF effort than would be expected by chance, are presented to summarize model results (Fig 2). Across all gears, both biophysical and governance variables were informative in predicting DWF effort. As expected, primary productivity is the most important biophysical variable in fixed gear and tuna purse seiner fisheries and ranks second in trawlers and third in longliners’ DWF. Nitrate is the only other biophysical informative variable across all fleets. At its 50m depth concentration, it ranks highest across all variables, biophysical and governance indicators, in trawler fisheries. Oxygen is an informative variable for fixed gear, trawlers, and tuna purse seiners. In terms of other biophysical factors, the fleets are very distinct. Governance effectiveness is the most important governance indicator in fixed gear, longliner and tuna purse seiner DWF, for the latter two it is the most informative across all variables. Control of corruption is the most important socio-political variable in trawler DWF fisheries.

**Fig 3:**
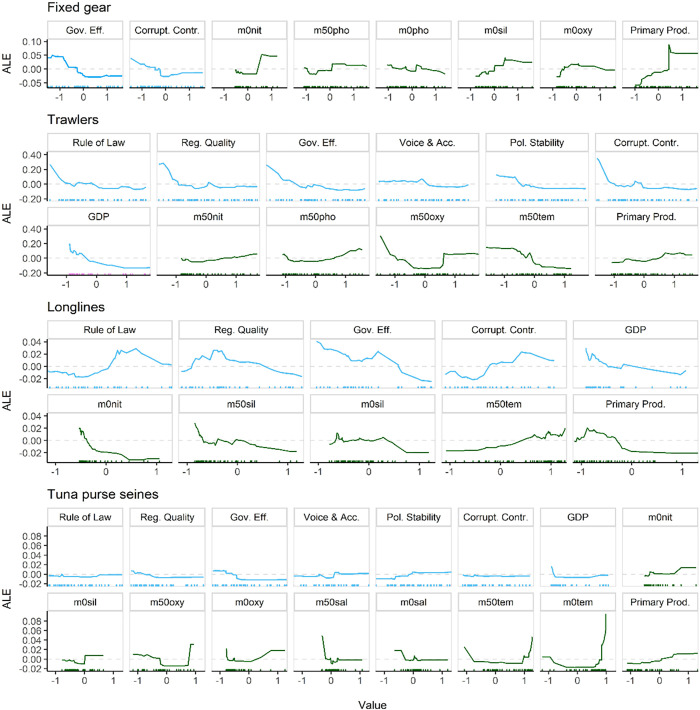
The influence of (normalized) environmental (green) and socio-political variables (blue) on DWF effort, as predicted by our random forest models and expressed as accumulated local effects (ALE), for the four fleets. Displayed are only informative variables (more important than a random variable, c.f. Fig 2) and for variable values within the 5 to 95 percentile intervals. Grey dashed lines indicate the 0 ALE level and bars along the x-axis indicate distribution of data points for this variable. Not all variables are more important than random for all four gears, therefore each panel may show different numbers of variables.

Fig 3 depicts, for each gear type, accumulated local effects (ALE) plots of all informative variables, i.e., more important than a random variable. ALE plots for all variables can be found in the supplementary material (Fig A5 in S1 File). Governance indicators are coded such that higher values indicate stronger and more effective governance. In trawler and fixed gear fisheries, all informative governance variables display a consistent negative relationship with DWF effort, with effort being higher when governance performance values are lower. This trend is alike for regulatory quality and governance effectiveness in purse seiners, as well as governance effectiveness and GDP in longliners. Other informative socio-political variables in purse seiners, and particularly longliners, show a different pattern. There is a slight tendency for higher purse seiner effort within states having higher political stability and voice and accountability. Longliner DWF effort peaks at intermediate ranges of rule of law, regulatory quality and shows a positive relationship with corruption control. Primary productivity is the only biophysical variable that is informative to all gear types, and effort is higher in more productive EEZs for fixed gear, trawler and purse seine fisheries, whereas effort for longliners peaks below mean primary productivity. Higher efforts of fixed gear fisheries co-occur with higher levels of silicate, oxygen, and nitrate. Phosphate concentrations increase at 50m depth, and rise at surface level with fixed gear DWF effort. Trawlers DWF efforts increase where primary productivity, nitrate and phosphate are high, oxygen is either particularly low or high, and temperature is low (all at 50m depth). Longliners operate at higher DWF effort where primary productivity, surface nitrate, and silicate at surface and 50m depth are low, and at highest efforts in the warmest EEZs. Tuna purse seine effort is higher with increasing primary productivity, surface nitrate, and temperature. Most DWF purse seining occurs in waters with below-average or very high oxygen concentrations, while above average surface silicate has positive effects. Salinity has very little effect on predicted tuna purse seine DWF effort except for peaks at very low salinities.

**Fig 4:**
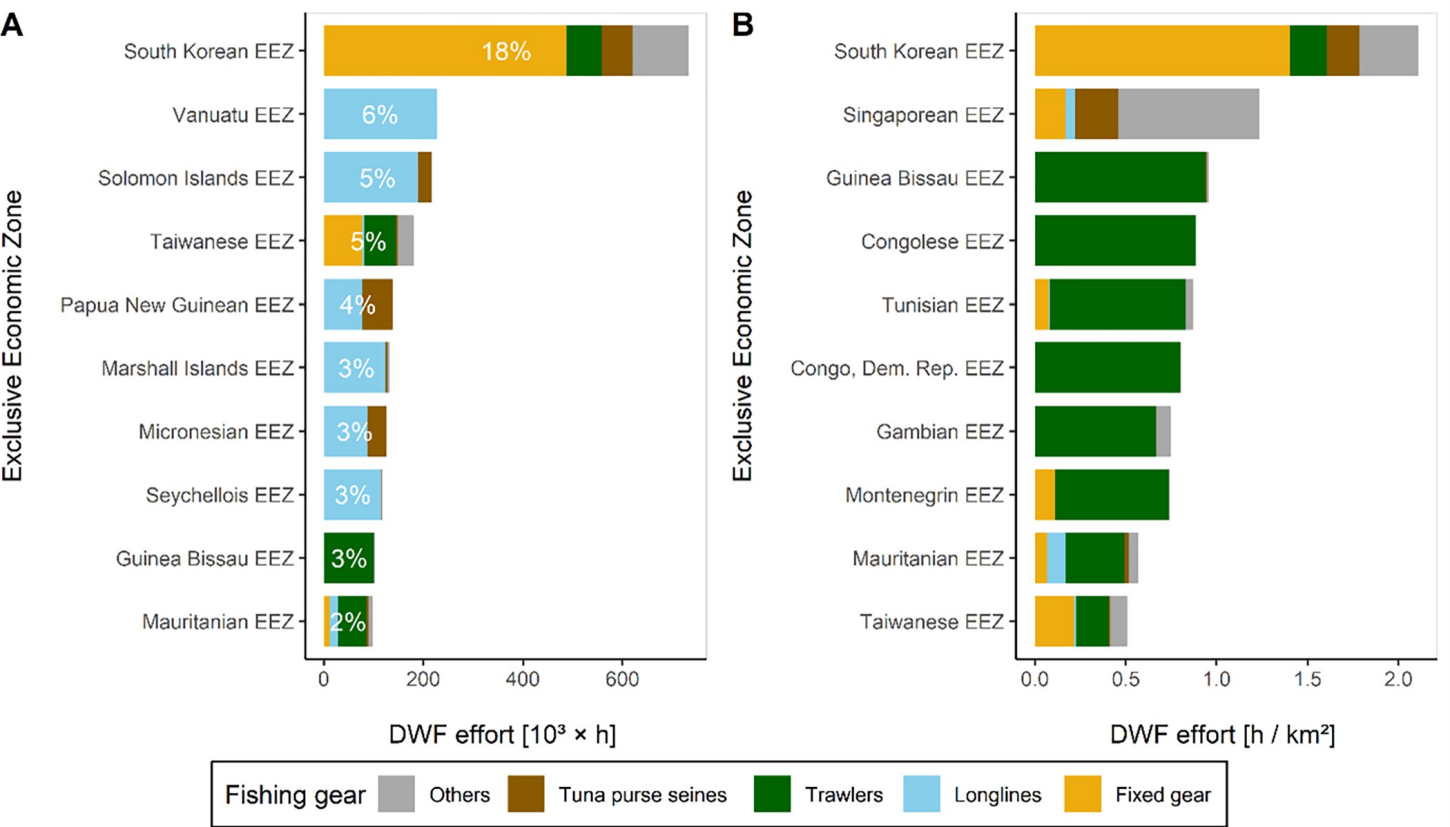
Aggregated distant water fishing effort (2017) by host countries’ exclusive economic zone (EEZ) as (A) total hours and (B) total hours divided by EEZ area. Numbers in the bars in chart A indicate the global percentage of the total distant water fishing effort received by each EEZ in 2017. Displayed are the 10 highest ranking EEZs, respectively.

As for summed DFW fishing hours, the top ten countries receive 52% of global DWF effort, and the top 5 EEZs account for 38% (Fig 4A). Effort in South Korean waters is by far the highest, driven by fixed gears. Longlining is responsible for high effort in many Pacific island states including Vanuatu, Solomon Islands, Papua New Guinea, Marshal islands, Micronesia, and the Seychelles in the Indian Ocean. However, when considering fishing density, i.e., dividing fishing hours by EEZ size (Fig 4B), west African and Mediterranean states have amongst the highest concentrations of DWF effort, largely driven by trawler fishing. South Korea again hosts the highest DWF effort densities, along with the Singaporean, Montenegrin and Taiwanese EEZs. Tuna purse seiner effort is highest in Papua New Guinean and South Korean EEZs, but does not dominate the total effort among any states in the way that longline and fixed gear effort does.

## 4.0 Discussion

Our results provide strong indications that governance performance plays a substantial role in determining how much distant water fishing (DWF) a host state receives. Even when biophysical parameters are considered, the historical assumption in fisheries science of ‘following the fish’ is a more nuanced reality when considering governance indicators in effort models. These results support the hypothesis that DWF effort for longline and tuna purse seine fleets is inversely related to governance performance, meaning fishing effort tends to increase as governance performance of the host state goes down. Variation across gear types and trend lines of individual indicators do vary. For fixed gear and trawlers, low governance performance is consistently associated with higher DWF effort.

The longline model follows the hypothesized pattern of increasing effort with decreasing governance indicator values except for the variables rule of law and control of corruption. There are many potential reasons for this. If the species targeted by longline fleets were already severely overexploited in countries with low rule of law and corruption control, fleets may have shifted into the waters of countries with higher governance indicators, resulting in the observed pattern of a higher density of effort in EEZs with better performance in corruption management and rule of law. As opposed to fixed gear or trawler vessels, longline fleets are observed to have high spatial flexibility due to targeting mobile oceanic species, such as tuna and swordfish, which are also highly valued and fished globally [50, 51]. This may provide additional incentive to pursue roving banditry, since quickly changing and rising fishing intensities exacerbates monitoring, control and surveillance of fishing fleets [52]. Thus, roving banditry practices may be able to avoid monitoring and sanctioning even under conditions of good rule of law and control of corruption, due to being further out to sea and conducting spatially sporadic practices. In contrast, it is likely more difficult for fixed gear and trawler fleets to fish further out to sea, as they tend to fish in more shallow waters, where monitoring and sanctioning may be more difficult to avoid. It may also be that host states are more willing to develop DWF fishing contracts for species that are fished with fixed gear or trawling techniques, or species of lower economic value. Such species may be less understood scientifically, or targeted by mostly by local small-scale fishers who generally have less political influence than industrial fishers in arguing for their own interests, and thus more vulnerable to elite capture.

Tuna purse seiners are more selective in their targeting behavior, while operating widely in tropical waters [17, 53]. Tuna purse seiners are the only gear type in which governance indicators dominantly exceed biophysical variables in their Variable Importance scores (Fig 2). However, no clear global patterns emerge in the direction in which these variables affect DWF effort, but instead effects appear to occur on smaller subranges along the values of the different governance indicators (Fig 3). This may reflect the complexity of governance and the interplay between national, regional, and sub-regional management bodies for tuna [54]. Purse seiners are usually large and technologically intensive vessels, targeting high value species. Findings from McCluney et al. [36] suggest that aggregate social, economic and governance performance of tuna fisheries is largely driven by market destination rather than regional management approaches, suggesting that the ability of governments to formulate and implement sound policies and regulations may be lacking in many areas where tuna are primarily fished. This may render them at greater influence on the complex governance attributes of tuna fleet host EEZ states.

In general, mobile oceanic species are often fished far offshore within EEZs and in variable locations requiring substantial investment into larger fleets with mechanized rigs, cold storage, onboard processing, and organized supply chains. This makes monitoring and sanctioning illegal fishing difficult because the species type and amount can be more easily hidden. Vessels, for example, could avoid going into local harbors, which altogether prevents host states’ ability to assess the state of their stocks based on species specific catch and quantity data with a sufficient degree of certainty [55]. Gaps in reporting and monitoring make the negotiation of DWF contracts less likely to be rooted in science-based assessments that reflect actual population status or ecosystem health, and less likely to account for illegal fishing. Literature on fishing contract agreements [21, 22, 24, 56] has indicated that they are often based on little knowledge of target fisheries status or value, and are often tied to or bundled together with other economic and political agreements between states. This may suggest that fishing contracts may not be the primary focus of a development or economic contract, but rather side bargaining entities that may leave the details of the gear type, location, landing and reporting obligations as secondary considerations if host states have the capacity to monitor those actions at all.

Governance effectiveness is the most informative governance indicator across all gears in our models, and control of corruption is a close second in fixed gear and trawler fisheries. It may be easier for fleets to exhibit strategies of roving banditry [52, 57] or to avoid detection of illegal fishing activities in the waters of states with lower scores in each. As hypothesized above, government effectiveness may reflect the ability of host states to develop sound DWF contracts with fair terms, for example based on accurate population assessments, market values or accounting for future ecosystem impacts. It may also be related to host states signing more generous contracts due to the lack of ability of their own domestic fleets to capitalize on their resources, or as can be further hypothesized, the knowledge of and the ability to capitalize on. For example, the value of fish on domestic markets may be lower than in host states, but host countries may not have access to international markets. For valuable oceanic species such as tuna, global markets may not differentiate where the fish was caught at all (i.e., consumer demand is blind to it), and thus, for them, the erosion of local fisheries is not problematic, because fish can be caught elsewhere to serve those markets.

Our analysis indicates that 5 of the top 10 countries with the highest spatial concentrations of DWF effort are west African states that are almost exclusively fished by trawling and to a minor share fixed gear, both of which are most strongly driven by governance effectiveness and control of corruption besides primary production. This is in line with Li and colleagues [58] find that DWF vessels are responsible for 60% of total fishing effort in African waters. Findings from Agnew et al., [8] show an inverse relationship between the World Governance Index scores and illegal, unregulated and unreported (IUU) fishing as a proportion of the total reported catch, and our findings support this with broader observational data. Governance effectiveness, as noted above, may indicate the ability of host states to develop fair DWF agreements that aren’t shadowed by the knowledge of the states conducting fishing that monitoring and sanctioning is unlikely, market value for certain species is higher internationally than locally, and that unknown population and ecosystem health assessments favor short-term intensive fishing effort (i.e., roving banditry). Corruption in the form of elite capture is a widespread issue in fisheries governance, and may be exacerbated in fishing contracts or illegal fishing if corruption in a country is generally problematic, including issues ranging from political bribery and organized crime [56] to supply chain transparency and slavery [25, 59]. A recent analysis McDonald et al., [60] used a predictive model and AIS data to identify over 75% of squid jigging (a fixed gear) and over 50% of longline vessels to be at high risk for using forced labor in 2017. These predicted forced-labor vessels spent less time in foreign EEZs and more time fishing in the open sea than vessels not classified as having forced labor [61, 62]. Labor issues are likely to be missing factors dictating the establishment of fishing contracts, interacting in the nexus between governance and effort, showing that including governance indicators in analyses, such as in this article, not only links to effort, but other important sustainability goals in fisheries.

IUU trawling is regularly observed, particularly in regions where governance may be lacking [23, 63] such as in African states dominated by distant water trawling [64]. Trawlers, for which we find a negative relationship between governance indices and DWF effort also employ forced labor, though proportionately less (under 5% of vessels in 2017) than longline and squid jigging vessels. However, unlike the other two fishing gear types, trawlers cannot operate their gear in the open sea. Despite this, research shows that trawlers are most likely to engage in transhipment at sea, followed by longliners, which can make fishing more efficient by avoiding frequent returns to port [65, 66]. This can create transparency issues linked to corruption and accountability because how much, where, and which species were caught is often unknown once fish are processed and packaged [65]. Transshipment facilities have also been associated human rights issues because the return less frequently to land and force laborers to remain onboard under dismal conditions [60, 66].

### 4.1 Fish grabbing

The term ‘fish grabbing’, draws from established land and water grabbing discourses [26, 28, 67, 68]. Grabbing narratives suggest that power disparities in natural resource and development acquisitions create asymmetric costs and benefits between states doing the grabbing (often those that have gained economic and political superiority through oppressive colonial histories, and thus have more information, technology and financial capital, and stronger domestic governance structures) and states being grabbed from (states with abundant natural resources, but often less economic and political power). Importantly, grabbing can still occur even if acquisition contracts are legal and jointly agreed upon, due to perhaps non-fisheries related economic and political dependencies such as foreign aid for development assistance, international political support, military alliances or pressure to follow a privatization based economic model. Our global findings stimulate various interpretations about what this means for specific fisheries in practice. The first being that countries with lower governance performance are making good use of their rights under the United Nations Convention of the Law of the Sea (UNCLOS), where they have the right to sell fishing rights to foreign countries to enable domestic economic gains even if they do not have their own capacity to fish. A more critical position may suggest that in the absence of strong pluralistic institutions domestically, elites could perform ‘fish grabbing’ from their own citizens, and sell rights under unfavorable terms which benefit elites but disadvantage citizens’ dependent on those fishery resources for livelihoods and food security. Fishing contracts may also be part of broader development oriented contracts and trades, negotiated in the context of owed debts, corporate lobbying, development aid or political maneuvering interests unrelated to fishing, but used as negotiable entities [22] as a form of elite capture. If neither of the above arguments explain our findings for a specific country, it suggests that a large majority of the effort is illegal, and thus a potentially large violation of sovereign rights.

A second argumentation is that lower governance performance suggests that a selected group of wealthier states, and fishing corporations flying their flags, are leveraging their economic and political power over states which have extensive, or underused fishery resources [21, 69]. Many countries with substantial fishery resources are located in the global tropics with emerging economies and political systems that may lack institutional leverage to negotiate fair contracts or monitor and enforce those contracts or illegal fishing activities. For host states, having a disadvantaged position in the process of setting contract terms may be due to various reasons including a lack of knowledge about the resources available and their value (e.g., stock assessments, foreign markets), economic dependencies on foreign capital or capacity for the process of negotiating itself. In their report on China’s DWF fleets, Gutierrez et al., [14] state that “economically weak countries in need of foreign currency, and without their own industrial fleets or scientific advice on sustainable catch limits, often negotiate disadvantageous fisheries agreements. Fragile governance and weak enforcement mean that low-income countries are also most at risk…, (p. 10).” In West Africa, patterns of illegal fishing and imbalance in resources appropriated versus value returned, holds for fishing contract relationships with the EU as well as with China [69]. These dynamics are likely well known to fishing states and corporations who have strong economic interests in trying to generate positive economic gains from their extensive subsidy inputs into distant water fishing, many of which are not profitable without them [3, 12]. Furthermore, the FAO’s (Food and Agriculture Organization of the United Nations) report on the State of the World Fisheries and Aquaculture (2018) [70] states that “the sustainability divide between developed and developing countries which has partially resulted from increased economic interdependencies, [is] coupled with limited management and governance capacity in developing countries,” (p. 5). If there is a generalizable divide resulting from the interdependencies between developed and developing states, it would be that developed countries have considerable leverage, or power, in dictating the terms and conditions of economic partnerships related to natural resource use, particularly as attention is now shifting towards Blue Growth and Blue Economy development strategies in many world regions.

A disadvantaged position in contract negotiations may not be necessary for explaining DWF increases in EEZs with low governance performances. If effort is increasing outside of contracts, this would be due to IUU fishing activity, which provides an opportunity to be observed with vessel Automated Identification System (AIS) data used in this analysis. However, AIS only records vessels with active devices and vessels who are consciously fishing illegally may be switching those off, which suggests that illegal fishing detected by AIS data is a conservative estimate. In this context, fish grabbing would be more of a strategic consideration based on where they are less likely to get caught due to a lack governance performance. If this is the case, then our findings may provide an explanation as to why IUU fishing occurs where it does, and where mitigation efforts could be targeted, such as in countries with weaker governance performance and highly productive waters.

More broadly, while a substantial literature on land and fresh water grabbing exists, only recently has marine resource grabbing emerged as a prominent issue [29–31]. Coastal areas are rapidly developing throughout the global tropics and many internationally driven economic development agendas have put Blue Growth and Blue Economy framings at the forefront of national development strategies [71–76]. An important argument within the grabbing discourse is that development strategies including resource use and development contracts with foreign states can often marginalize local communities who depend on those resources but are not empowered to voice their needs and concerns (i.e., through corruption, elite capture). Such contracts may attract political and economic elites seeking foreign capital and infrastructure investment, but the degree to which those development strategies serve the needs of local citizens needs to be raised. These issues are particularly salient in the conflicts between small-scale fisheries and industrial foreign fleets. Foreign fleets do not have to bear the economic, environmental or management costs of ecosystem degradation or fish population declines in the way that local and livelihood dependent small-scale fishers do, although they may often compete for the same fish in the same waters. Small-scale fishing communities are often located in rural areas and may not have the economic or educational opportunities that could empower them to have a voice in political decision making on fisheries issues. While host states may receive monetary compensation from foreign fishing fleets, even if that compensation does make its way back to communities directly impacted, access to the nutritional value of fish taken by DWF is removed for local populations, where it is oftentimes urgently needed to avoid malnutrition and safeguard food security [64, 77].

### 4.2 Methodological considerations

Although the GFW algorithms identifying fishing effort from raw ship position (AIS) data are very precise [17], the largest flaws remain the reception capacity of AIS signals broadcasted by ships and the fact that it may be switched off. Recent studies have complemented AIS data with additional information and fishing fleets not represented in AIS data [78, 79] and shown that terrestrial receivers perform better at capturing all AIS signals than satellites [80]. Given these details, our findings of DWF densities are likely an underestimation and may be interpreted as conservative, especially in low-income countries where there are fewer terrestrial receivers.

Our findings represent a general trend, and the meaning for any specific country needs to be considered in the social, economic, and environmental context in which illegal fishing or power imbalances in negotiating foreign fishing contracts may occur. The World Governance Indicators (WGI) [43] provide generalized indications of a state’s governance performance, but they are not specific to the fisheries sector, although they have provided enough signal for meaningful explanatory power in our models. This is supported by Davies et al., [81] who show that the WGI is “strongly associated with marine aquaculture governance features” (p. 31) and can be used for further analyses in the sector. Agnew et al., [8] show similar findings with an inverse relationship between the WGI and the amount of illegal fishing at the country level. WGI data is only available at annual intervals.

Our models are built per year, while Crespo [82] found that monthly resolved models predicting high sea pelagic longline fishing effort outperformed models aggregating data over a year, and that their performance metrics were statistically dissimilar. To counteract the large variety in EEZs sizes, we standardized DWF effort per EEZ area, which is crucial for a global comparison. However, in some cases, this led to high standardized DWF effort in small EEZs like Singapore and Montenegro, which might blur our analysis to a small extend.

## 5.0 Conclusion

Our findings indicate that consideration of both social and ecological performance indicators in models explaining distant water fishing (DWF) effort is needed to gain an accurate understanding of the many potential factors shaping observed vessel behavior. Global fishing effort data quality and quantity will continue to improve, and studies continuing to examine the relationship between governance performance and effort is encouraged, over both space and time. Importantly, our findings suggest that governance performance indicators can be more important in explaining effort in some DWF gear or species specific fleets such as tuna than traditionally used ecological factors such as primary productivity. Despite the many nuances regarding why and to what extent, our findings suggest an observed general trend that host states tend to receive more DWF fishing effort from foreign fleets if they perform lower on governance performance indicators. We have suggested that the narrative of ‘grabbing’ may be useful for framing explanations of why often less economically and political powerful host states with abundant fisheries resources are seemingly more exploited by foreign fleets who have gained power and influence through often oppressive colonial and imperialistic practices. Many states with large DWF fleets know in which states they are less likely to be sanctioned or monitored, or have enough political and economic influence over certain host states to shape fishing agreements in their favor, perhaps due to the lack of the host state’s knowledge of their own full value and population status or costs of degradation. In practice, a large knowledge gap in DWF analysis is gaining access to the details of fishing agreements between states, to ensure that such agreements uphold commitments to UNCLOS, as well as to understand who benefits from agreements, and to be able to separate illegal from legal DWF effort in observational data.

## Supporting information

S1 File (DOCX)
