# Supplementary material for: Fish grabbing: Weak governance and productive waters are targets for distant water fishing
Source: PLoS One. 2022 Dec 6;17(12):e0278481. doi: 10.1371/journal.pone.0278481 (PMC9725136; doi:10.1371/journal.pone.0278481)
Supplement: S1 File — (DOCX) [file pone.0278481.s001.docx]

**Supplementary Information for**

Fish grabbing: weak governance and productive waters are targets for distant water fishing

Moritz Stäbler ^1§^, Jonas Letschert ^2§^, Marie Fujitani ^1,3^, Stefan Partelow ^1,^ *

1) Leibniz Centre for Tropical Marine Research (ZMT), Germany

2) Thuenen Institute of Sea Fisheries, Germany

3) University of Bremen, Germany

* Corresponding author: Stefan Partelow

§ Stäbler and Letschert share first author role

**Email:**  [stefan.partelow@leibniz-zmt.de](mailto:stefan.partelow@leibniz-zmt.de)

**This PDF file includes:**

Figures A1, A2, A3, A4, A5, A6

Tables S1, S2


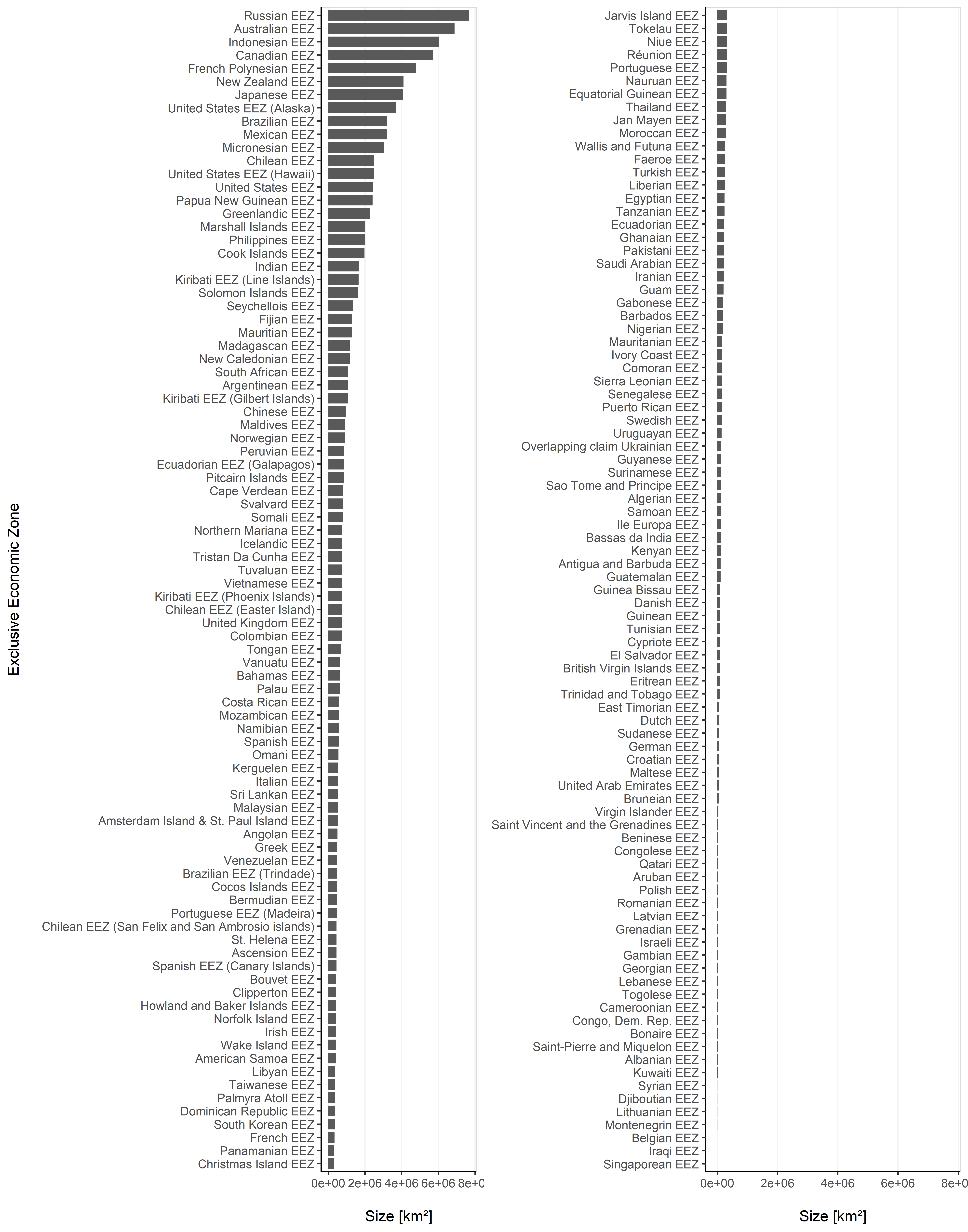


**Figure A1.** EEZs with DWF occurring in 2017, ranked by size.


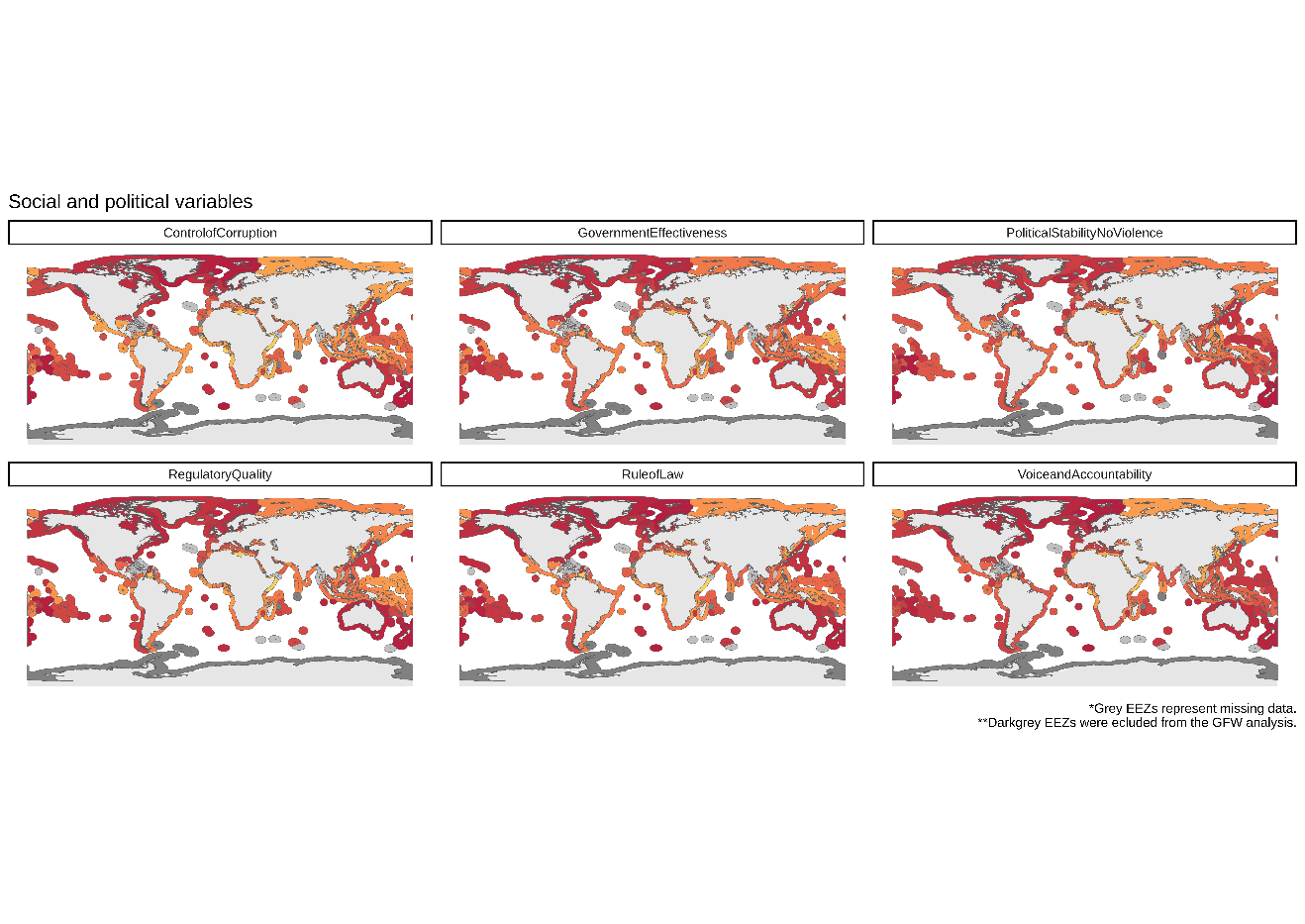


**Figure A2.** Governance variable value distribution across Exclusive Economic Zones (EEZs) considered in this analysis. All values are normalized to range of 0 to 1.


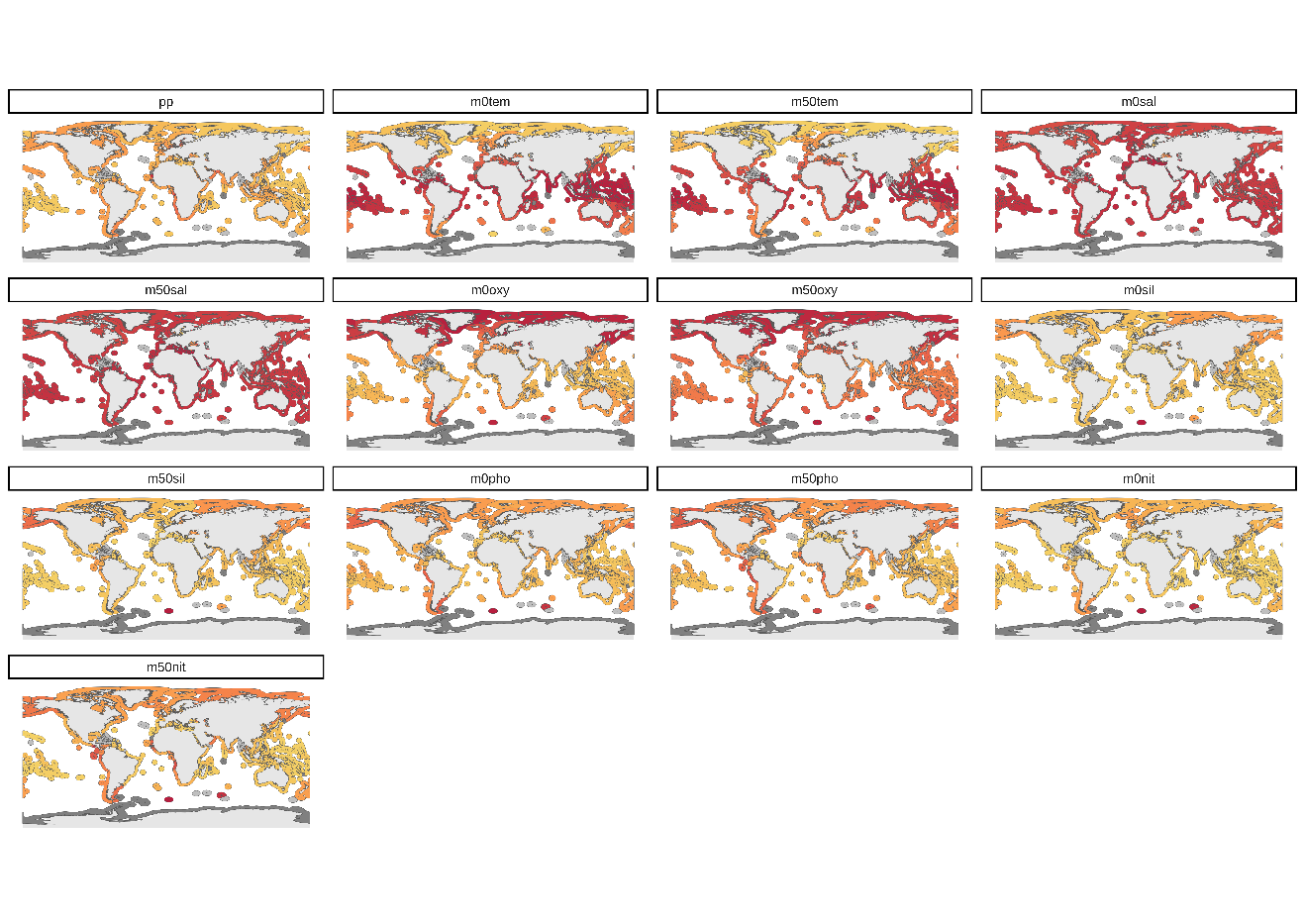


**Figure A3.** Biophysical variable value distribution across Exclusive Economic Zones (EEZs) considered in this analysis. All values are normalized to range of 0 to 1.


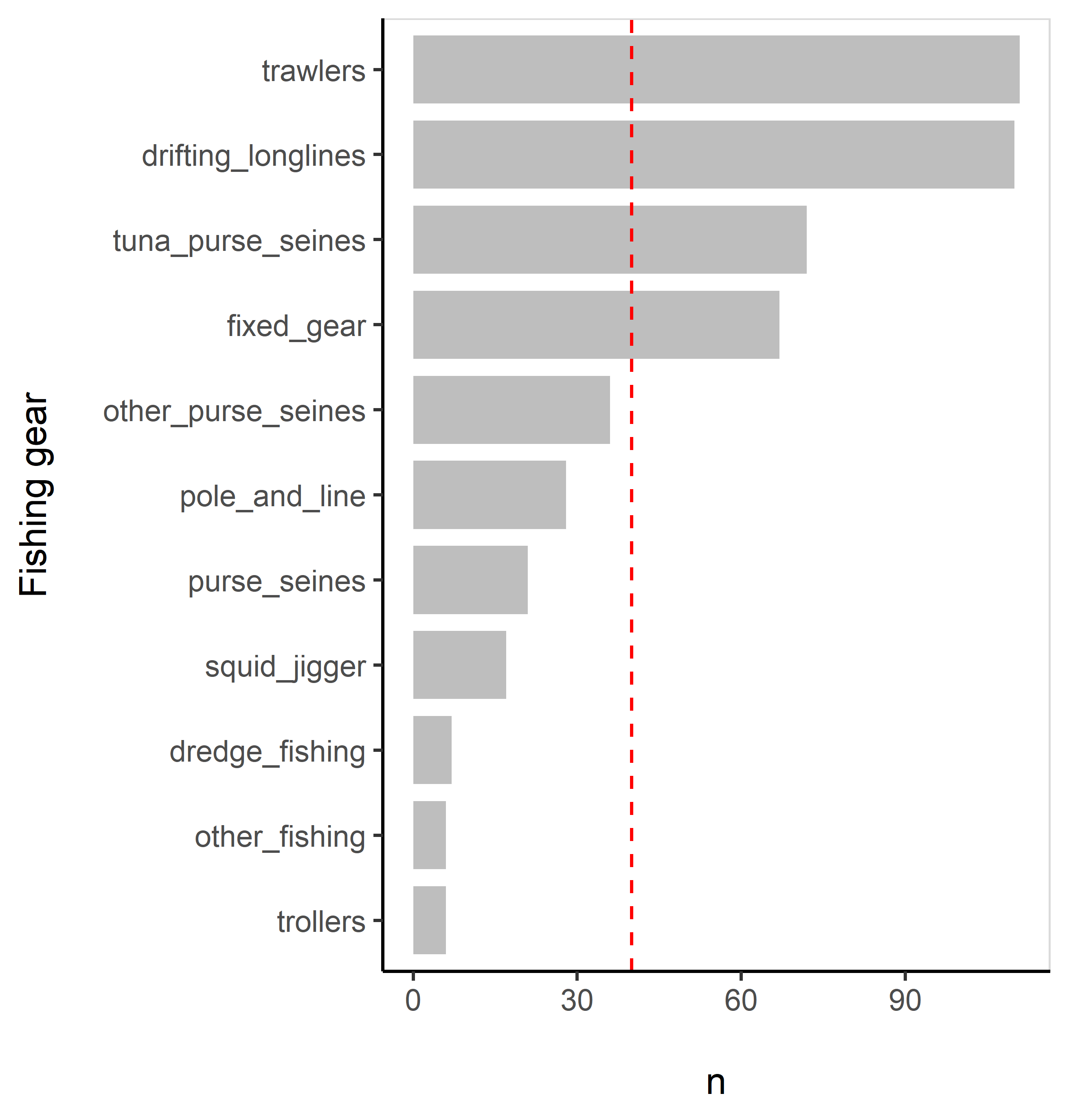


Figure A4. Sample size, as number of exclusive economic zones, in which distant water fishing occurred in 2017. We only included gear types occurring in 40 EEZs or more into our modelling.

Table S1. Exclusive economic zone (EEZ) removed from the analysis. We removed fishing effort in case the flag state was equal to the sovereignty of an EEZ (*State territory*). We removed the entire EEZ from the analysis in case of *disputed* EEZs, *joint* regimes, or *no sovereignty* or if there were no available information for primary production (*No* *PP*). Note that the baseline for this table are EEZs in which distant water fishing effort occurred in 2017.

| Sovereignty | Exclusive Economic Zone | State territory | Disputed | Joint regime | No sovereignty | No PP |
| --- | --- | --- | --- | --- | --- | --- |
| - | Antarctic 200NM zone beyond the coastline |  |  |  | X |  |
| Australia | Cocos Islands EEZ | X |  |  |  |  |
| Australia | Christmas Island EEZ | X |  |  |  |  |
| Australia | Heard and McDonald Islands EEZ | X |  |  |  |  |
| Australia | Norfolk Island EEZ | X |  |  |  |  |
| Brazil | Brazilian EEZ (Trindade) | X |  |  |  |  |
| Multiple | Overlapping claim: Canada / USA |  | X |  |  |  |
| Chile | Chilean EEZ (Easter Island) | X |  |  |  |  |
| Chile | Chilean EEZ (San Felix and San Ambrosio islands) | X |  |  |  |  |
| Multiple | Joint regime area Costa Rica / Ecuador (Galapagos) |  |  | X |  |  |
| Denmark | Faeroe EEZ | X |  |  |  |  |
| Denmark | Greenlandic EEZ | X |  |  |  |  |
| Multiple | Joint regime area Colombia / Dominican Republic |  |  | X |  |  |
| Multiple | Joint regime area Ecuador / Colombia |  |  | X |  |  |
| Multiple | Overlapping claim Doumeira Islands: Djibouti / Eritrea |  | X |  |  |  |
| France | Crozet Islands EEZ | X |  |  |  |  |
| France | Amsterdam Island & St. Paul Island EEZ | X |  |  |  |  |
| France | Bassas da India EEZ | X |  |  |  |  |
| France | Ile Europa EEZ | X |  |  |  |  |
| France | Juan de Nova EEZ | X |  |  |  |  |
| France | Kerguelen EEZ | X |  |  |  |  |
| France | Saint-Barthélemy EEZ | X |  |  |  |  |
| France | Clipperton EEZ | X |  |  |  |  |
| Multiple | Joint regime area Spain / France |  |  | X |  |  |
| Multiple | Joint regime area Italy / France |  |  | X |  |  |
| France | Guadeloupean EEZ | X |  |  |  |  |
| France | French Guiana EEZ | X |  |  |  |  |
| France | Saint-Martin EEZ | X |  |  |  |  |
| France | Martinican EEZ | X |  |  |  |  |
| Multiple | Overlapping claim Mayotte: France / Comoros |  | X |  |  |  |
| France | New Caledonian EEZ | X |  |  |  |  |
| Multiple | Overlapping claim Matthew and Hunter Islands: New Caledonia / Vanuatu |  | X |  |  |  |
| France | French Polynesian EEZ | X |  |  |  |  |
| France | Réunion EEZ | X |  |  |  |  |
| Multiple | Overlapping claim Ile Tromelin: Reunion / Mauritus |  | X |  |  |  |
| France | Saint-Pierre and Miquelon EEZ | X |  |  |  |  |
| France | Wallis and Futuna EEZ | X |  |  |  |  |
| Multiple | Overlapping claim: Trinidad and Tobago / Venezuela / Guyana |  | X |  |  |  |
| Multiple | Overlapping claim Navassa Island: USA / Haiti |  | X |  |  |  |
| Multiple | Joint regime area Iceland / Norway (Jan Mayen) |  |  | X |  |  |
| Multiple | Overlapping claim Kuril Islands: Japan / Russia |  | X |  |  |  |
| Multiple | Overlapping claim Liancourt Rocks: Japan / South Korea |  | X |  |  |  |
| Multiple | Overlapping claim: Kenya / Somalia |  | X |  |  |  |
| Multiple | Overlapping claim Glorioso Islands: France / Madagascar |  | X |  |  |  |
| Netherlands | Aruban EEZ | X |  |  |  |  |
| Netherlands | Sint-Eustatius EEZ | X |  |  |  |  |
| Netherlands | Bonaire EEZ | X |  |  |  |  |
| Netherlands | Curaçaoan EEZ | X |  |  |  |  |
| Netherlands | Sint-Maarten EEZ | X |  |  |  |  |
| Netherlands | Saba EEZ | X |  |  |  |  |
| New Zealand | Cook Islands EEZ | X |  |  |  |  |
| New Zealand | Niue EEZ | X |  |  |  |  |
| New Zealand | Tokelau EEZ | X |  |  |  |  |
| Norway | Bouvet EEZ | X |  |  |  |  |
| Multiple | Joint regime area Sweden / Norway |  |  | X |  |  |
| Norway | Jan Mayen EEZ | X |  |  |  |  |
| Norway | Svalvard EEZ | X |  |  |  |  |
| Multiple | Joint regime area Peru / Ecuador |  |  | X |  |  |
| Portugal | Portuguese EEZ (Azores) | X |  |  |  |  |
| Portugal | Portuguese EEZ (Madeira) | X |  |  |  |  |
| Multiple | Joint regime area Nigeria / Sao Tome and Principe |  |  | X |  |  |
| Multiple | Joint regime area Senegal / Guinea Bissau |  |  | X |  |  |
| Multiple | Joint regime area Japan / Korea |  |  | X |  |  |
| Multiple | Overlapping claim Ceuta: Spain / Morocco |  | X |  |  |  |
| Spain | Spanish EEZ (Canary Islands) | X |  |  |  |  |
| Multiple | Overlapping claim: Sudan / Egypt |  | X |  |  |  |
| Multiple | Overlapping claim Senkaku Islands: Japan / China / Taiwan |  | X |  |  |  |
| Multiple | Overlapping claim: Iran / United Arab Emirates |  | X |  |  |  |
| United Kingdom | Anguilla EEZ | X |  |  |  |  |
| United Kingdom | Ascension EEZ | X |  |  |  |  |
| United Kingdom | Bermudian EEZ | X |  |  |  |  |
| United Kingdom | Cayman Islands EEZ | X |  |  |  |  |
| Multiple | Overlapping claim Falkland / Malvinas Islands EEZ: UK / Argentina |  | X |  |  |  |
| Multiple | Joint regime area United Kingdom / Denmark (Faeroe Islands) |  |  | X |  |  |
| United Kingdom | Guernsey EEZ | X |  |  |  |  |
| Multiple | Overlapping claim Gibraltarian EEZ |  | X |  |  |  |
| Multiple | Overlapping claim Chagos Archipelago EEZ: UK / Mauritius |  | X |  |  |  |
| United Kingdom | Jersey EEZ | X |  |  |  |  |
| United Kingdom | Montserrat EEZ | X |  |  |  |  |
| United Kingdom | Pitcairn Islands EEZ | X |  |  |  |  |
| Multiple | Overlapping claim South Georgia and South Sandwich EEZ: UK / Argentina |  | X |  |  |  |
| United Kingdom | St. Helena EEZ | X |  |  |  |  |
| United Kingdom | Tristan Da Cunha EEZ | X |  |  |  |  |
| United Kingdom | Turks and Caicos EEZ | X |  |  |  |  |
| United Kingdom | British Virgin Islands EEZ | X |  |  |  |  |
| United States | American Samoa EEZ | X |  |  |  |  |
| United States | Guam EEZ | X |  |  |  |  |
| United States | Northern Mariana EEZ | X |  |  |  |  |
| Multiple | Overlapping claim: Puerto Rico / Dominican Republic |  | X |  |  |  |
| United States | Puerto Rican EEZ | X |  |  |  |  |
| United States | Johnston Atoll EEZ | X |  |  |  |  |
| United States | Howland and Baker Islands EEZ | X |  |  |  |  |
| United States | Jarvis Island EEZ | X |  |  |  |  |
| United States | Wake Island EEZ | X |  |  |  |  |
| United States | Palmyra Atoll EEZ | X |  |  |  |  |
| United States | Virgin Islander EEZ | X |  |  |  |  |
| Multiple | Joint regime area Argentina / Uruguay |  |  | X |  |  |
| Multiple | Overlapping claim Western Saharan EEZ |  | X |  |  |  |
| Monaco | Monegasque EEZ |  |  |  |  | X |

**Table S2.** Data categories, sources and specific indicators used.

| **Data category** | **Data source** | **Specific indicators** |
| --- | --- | --- |
| Fishing effort | Global Fishing Watch (GFW) <https://globalfishingwatch.org/> (accessed 01.03.2019) | Annual fishing effort in hours in each host EEZ aggregated by sending state and split into the six GFW gear type categories. Further, effort density was calculated by dividing it by the size of the EEZ in km². |
| Governance performance | Worldwide Governance Indicators (WGI)  <https://info.worldbank.org/governance/wgi/> | Control of Corruption |
|  |  | Government Effectiveness |
|  |  | Political Stability (No Violence) |
|  |  | Regulatory Quality |
|  |  | Rule of Law |
|  |  | Voice and Accountability |
| Economic performance | World Bank  [https://data.worldbank.org/](https://data.worldbank.org/indicator/NY.GDP.PCAP.CD) | Gross Domestic Product (GDP) per capita from 2017 in USD |
| Fisheries productivity | Seas Around Us [http://www.seaaroundus.org/](http://www.seaaroundus.org/sea-around-us-area-parameters-and-definitions/#_Toc421807913) | Primary productivity |
|  | NOAA World Ocean Atlas  <https://www.nodc.noaa.gov> | Temperature (SST) |
|  |  | Temperature (50m) |
|  |  | Salinity (SST) |
|  |  | Salinity (50m) |
|  |  | Oxygen concentration (SST) |
|  |  | Oxygen concentration (50m) |
|  |  | Nitrate (SST) |
|  |  | Nitrate (50m) |
|  |  | Phosphate (SST) |
|  |  | Phosphate (50m) |


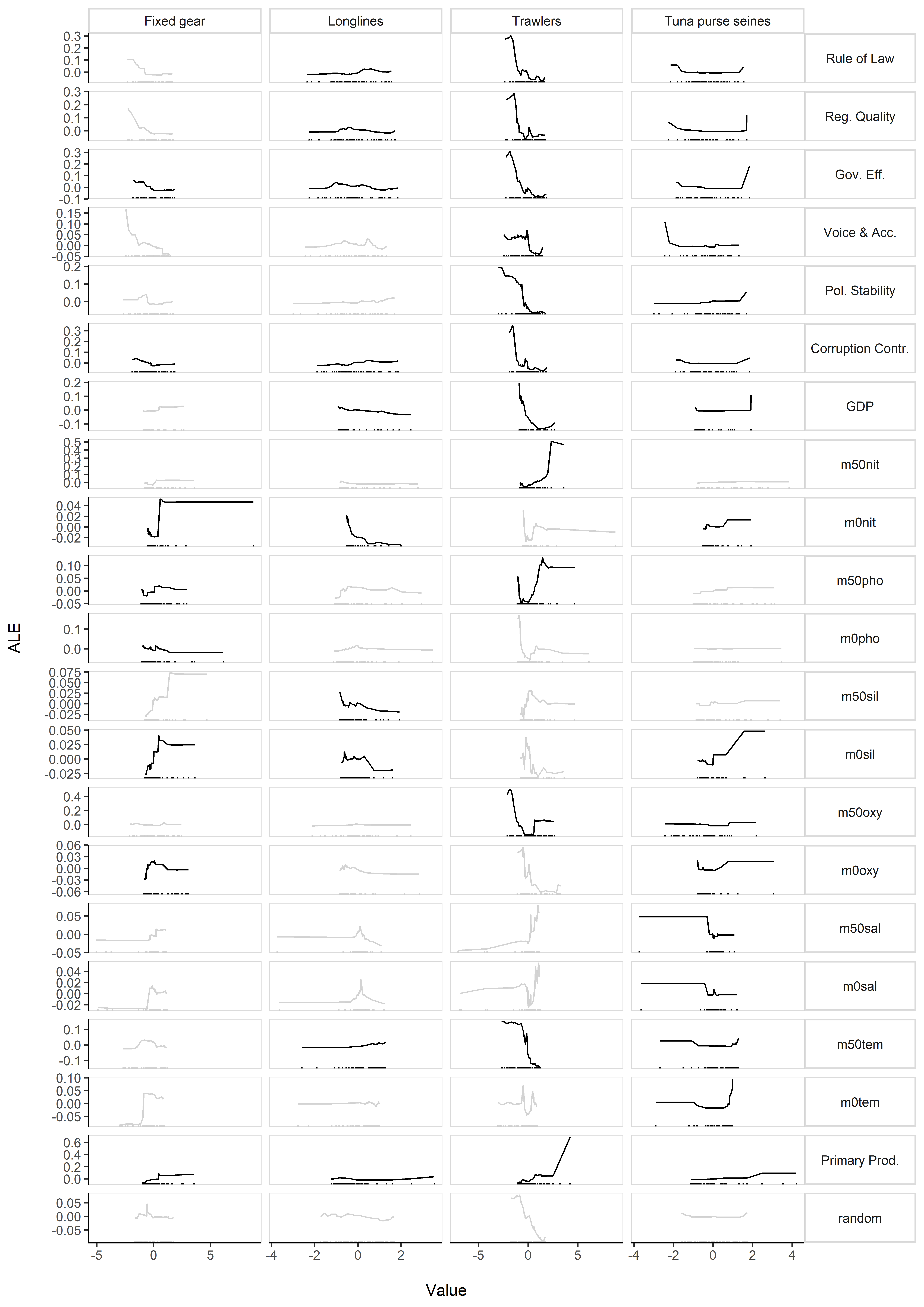


**Figure A5.** Accumulated Local Effects (ALE) plots for all *(A)* environmental and *(B) socio-economic variables* of all four fishing gears. Variables with Variable Importances (VI) higher than that of a random variable (c.f. Figure 2, Figure 3) are represented in black, other by grey lines.


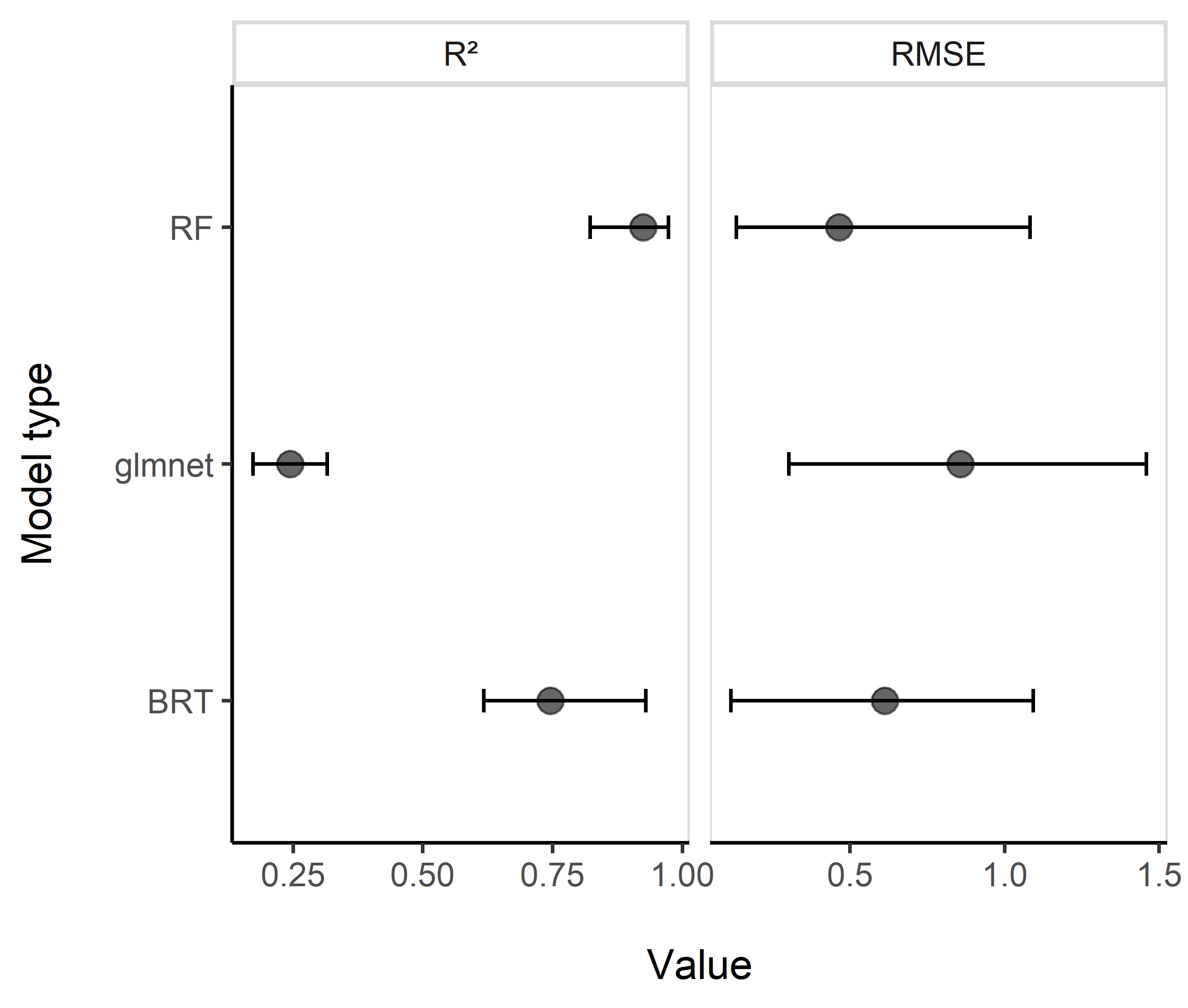


Figure A6. Model performances of the three model families evaluated for the four different gear types. Bars represent lowest and highest, and the dot mean values.
